# Cardioaortic dimensions in German landrace pigs derived from cardiac magnetic resonance imaging

**DOI:** 10.1038/s41598-024-52376-x

**Published:** 2024-01-22

**Authors:** Florian Meissner, Michelle Costa Galbas, Sophie Szvetics, Constantin von zur Mühlen, Timo Heidt, Alexander Maier, Michael Bock, Martin Czerny, Wolfgang Bothe, Simon Reiss

**Affiliations:** 1 Department of Cardiovascular Surgery, University Heart Center Freiburg – Bad Krozingen, Faculty of Medicine, University of Freiburg, Hugstetter Strasse 55, 79106 Freiburg, Germany; 2 Department of Cardiology and Angiology, University Heart Center Freiburg – Bad Krozingen, Faculty of Medicine, University of Freiburg, Hugstetter Strasse 55, 79106 Freiburg, Germany; 3 Department of Diagnostic and Interventional Radiology, Medical Physics, Faculty of Medicine, University of Freiburg, Killianstrasse 5a, 79106 Freiburg, Germany

**Keywords:** Cardiac device therapy, Interventional cardiology, Preclinical research, Anatomy, Cardiovascular diseases

## Abstract

Pigs are frequently applied as animal models in cardiovascular research due to their anatomical and physiological similarity to humans. For study planning and refinement, precise knowledge of the cardioaortic dimensions is essential. In a retrospective single-center study, the cardioaortic dimensions and left ventricular function of German Landrace pigs were assessed using cardiac MRI. All parameters were compared between male and female pigs and analyzed for correlation with body weight. In total, 15 pigs were included (7 male and 8 female, weight 60.9 ± 7.0 kg). The left ventricle revealed an end-diastolic diameter of 50.5 ± 4.4 mm and an ejection fraction of 51.2 ± 9.8%. The diameters of the ascending and descending aorta were 21.3 ± 2.3 and 16.2 ± 1.4 mm, respectively. There were no significant differences between male and female pigs, except that males had a smaller end-diastolic left ventricular volume (*p* = 0.041). A moderate correlation was found between body weight and the aortic annulus diameter (*R* = 0.57, *p* = 0.027). In conclusion, cardiac MRI allows precise quantification of porcine cardioaortic dimensions. For medical device testing, size differences between pigs and humans should be considered.

## Introduction

Domestic pigs (*Sus scrofa domesticus*) are commonly used as large animal models in experimental cardiovascular research^1^. They are applied for pre-clinical studies performing coronary catheterization^2^, left ventricular assist device (LVAD)^3^ and transcatheter valve implantation (TAVI)^4^, as well as catheter device development^5,6^. Despite many similarities between human and porcine anatomy and physiology, relevant differences remain between both species. Exemplarily, Crick et al*.* describe the classic Valentine shape of the porcine heart, whereas the human one is more trapezoidal. In pigs, the cranial and caudal vena cava enter the right atrium not in line but nearly right-angled^7^. Compared to humans, pigs reveal smaller atria and a more prominent hemiazygos vein, which enters the coronary sinus anterior to the left pulmonary vein. Furthermore, the porcine left common carotid artery arises from the brachiocephalic artery (BCA) instead of the aortic arch, which generally has only two supra-aortic branches^8^.

A comprehensive understanding of these differences is crucial for study planning and refinement, helping to comply with the *3R principles* (replacement, reduction, refinement). Moreover, study- and application-specific differences need to be considered for animal testing. For TAVI procedures, particularly vascular access and coronary ostia heights are relevant. For LVAD implantation, the dimensions of the left ventricle (LV) and its wall thickness are of interest.

Different imaging modalities are applied to plan cardiovascular procedures. Since transthoracic echocardiography (TTE) appears to be challenging in growing pigs due to their keel-shaped thorax and thicker sternum, the overlying lungs at the midline, and their prominent body fat, only transesophageal echocardiography (TEE) allows comprehensive non-invasive echocardiographic assessment^8^. It helps anesthesiologists to guide cardiovascular procedures and supports decision-making. Despite many advantages, TEE is technically complex and requires dedicated equipment, advanced training, and expertise. Furthermore, probe placement may be limited by anatomical restrictions and is associated with a few severe and potentially fatal complications (e.g., upper gastrointestinal injuries)^9^. Instead, computed tomography angiography (CTA) is used for morphological assessment, for example, to plan TAVI procedures in pigs^4^ and porcine-to-human xenotransplantations^8^. It is also the means of choice for imaging after the implantation of certain medical devices, which are incompatible with cardiac magnetic resonance imaging (cMRI), allowing an optimal before-and-after comparison. Although CTA is associated with radiation exposure, which has decreased significantly over the past years, it has many advantages, including its short acquisition time of a few minutes. Instead, cMRI can be used for functional assessment, for example, to determine the LV systolic function. Due to its high spatial resolution and soft tissue contrast, cMRI is considered the gold standard for heart tissue imaging and ventricular volumetry^10^. Unfortunately, MRI is more expensive and requires a much longer acquisition time than CTA.

Depending on the study purpose and the availability of advanced imaging modalities, different parameters may be assessed. Referring to our research fields, mechanical circulatory support devices and coronary interventions, we are interested in specific cardioaortic dimensions. Among others, we develop an LVAD accessory redirecting the blood from the pump through a transventricular outflow graft across the aortic valve (AV) into the ascending aorta (AAo)^3,11^. In contrast, conventional devices direct the blood through an extraventricular outflow graft anastomosed to the AAo. To define the transventricular outflow length, it is essential to be aware of the distances between the LV apex and AV, sinotubular junction (STJ), and BCA. Since the outflow graft may be implanted either transapically or transfemorally using a customized delivery system, detailed information on the vascular access, diameters, and angulations is required to define the size and stiffness of the delivery system. Such data should be obtained from animals of the same breed and body weight (BW), like those scheduled for pre-clinical testing. In some cases, size differences between humans and pigs require adjustments to the device design.

This study assessed selected cardioaortic dimensions from German Landrace pigs undergoing cMRI-guided coronary catheterization—to support study planning and help researchers refine medical device testing. Moreover, we aimed to compare male and female pigs and to determine the correlation between BW and cardioaortic dimensions.

## Methods

### Animal study

In a single-center study at the University Medical Center Freiburg, Germany, the cardioaortic dimensions of 15 German Landrace pigs (7 castrated male and 8 female, weight 60.9 ± 7.0 kg) were assessed from cMRI scans obtained between March 2021 and February 2023. The scans were acquired in an animal study investigating myocardial reperfusion after temporary coronary occlusion^2^. All experiments were approved by the local animal welfare committee (Regierungspräsidium Freiburg, Germany, G-21/008). The research was conducted in accordance with the German animal protection law (TierSchG), the European Convention for the Protection of Vertebrate Animals used for Experimental and other Scientific Purposes, and the ARRIVE guidelines^12,13^. All pigs were accommodated in the experimental animal facility of the University of Freiburg and under the care of experienced staff veterinarians. The pigs were fed a standard pellet chow (20 g/kg) and had access to water ad libitum. All animals were kept under controlled environmental conditions at 20 °C, 75 ± 5% humidity, and a 13/11-h light/dark cycle.

### Anesthesia

After preoxygenation, anesthesia was induced with propofol (2‒4 mg/kg IV) and maintained with a mixture of isoflurane (1.5‒2.0%) and oxygen/air (FiO_2_ 30‒40%) as well as vecuronium (0.2‒0.4 mg/kg/h IV) and fentanyl (0.002‒0.004 mg/kg/h IV). Mechanical ventilation was adjusted to maintain physiological conditions. Peri-interventional monitoring included electrocardiography, pulse oximetry, arterial blood pressure, and capnography. Body temperature was determined before and after cMRI. All animals were covered with a blanket without external heat support. For fluid substitution, an isotonic saline solution (5‒10 mL/kg IV) was administered.

### Coronary intervention

The coronary intervention was performed using standard clinical catheters, which were inserted transfemorally (6 F). To reduce the risk of cardiac arrhythmias, a single dose of amiodarone (10 mg/KG IV) was administered prior to coronary manipulation. Under angiographic guidance, the left coronary artery (LCA) was intubated, and a coronary wire (0.018 inches) was placed in the left circumflex coronary. An occlusive balloon was inflated in the mid-coronary segment to induce myocardial ischemia. The balloon was removed after 40 min.

### MRI imaging protocol and data reconstruction

After removal of the occlusive balloon catheter, cMRI was performed at a clinical 3 T system (PrismaFit, Siemens, Erlangen, Germany). There was no pre-ischemic imaging providing a baseline. All animals were examined in a supine position, under general anesthesia with mechanical ventilation, and with the heart at the magnet’s isocenter. A posterior 32-channel spine coil and an anterior 18-channel thorax coil array were used for signal reception. Three imaging sequences were used to assess the cardioaortic geometry. First, a 3D compressed-sensing accelerated whole heart navigator- and electrocardiography-gated prototype FLASH sequence (provided by the system vendor) was acquired. The acquisition parameters included an echo/repetition time (TE/TR) of 2.3/5.2 ms, a field of view (FoV) of 310 × 320 × 130 mm^3^, an in-plane resolution of 1.25 × 1.25 × 1.25 mm^3^, a flip angle of (α) 15°, an acceleration factor (R) of 6.4, and a bandwidth of 250 Hz/px with fat saturation and T_2_-preparation. For functional imaging, a multi-slice 2D bSSFP CINE sequence was acquired in short-axis orientation during manually induced breath-hold (TE/TR: 1.5/3.3 ms, FoV: 340 × 273 mm^2^, resolution: 1.52 × 2.17 mm^3^, slice thickness: 8 mm, α = 42°, R = 3, bandwidth: 970 Hz/px). The length of the aortic arch was measured on real-time 2D FLASH images covering the aortic arch (TE/TR: 1.3/2.8 ms, FoV: 289 × 289 mm^2^, resolution: 1.5 × 2.0 mm^3^, slice thickness: 8 mm, α = 10°, R = 2, BW: 790 Hz/px, fat saturation). Further details of the experiments can be found in previous publications^14–16^.

### Data analysis

**Figure 1:**
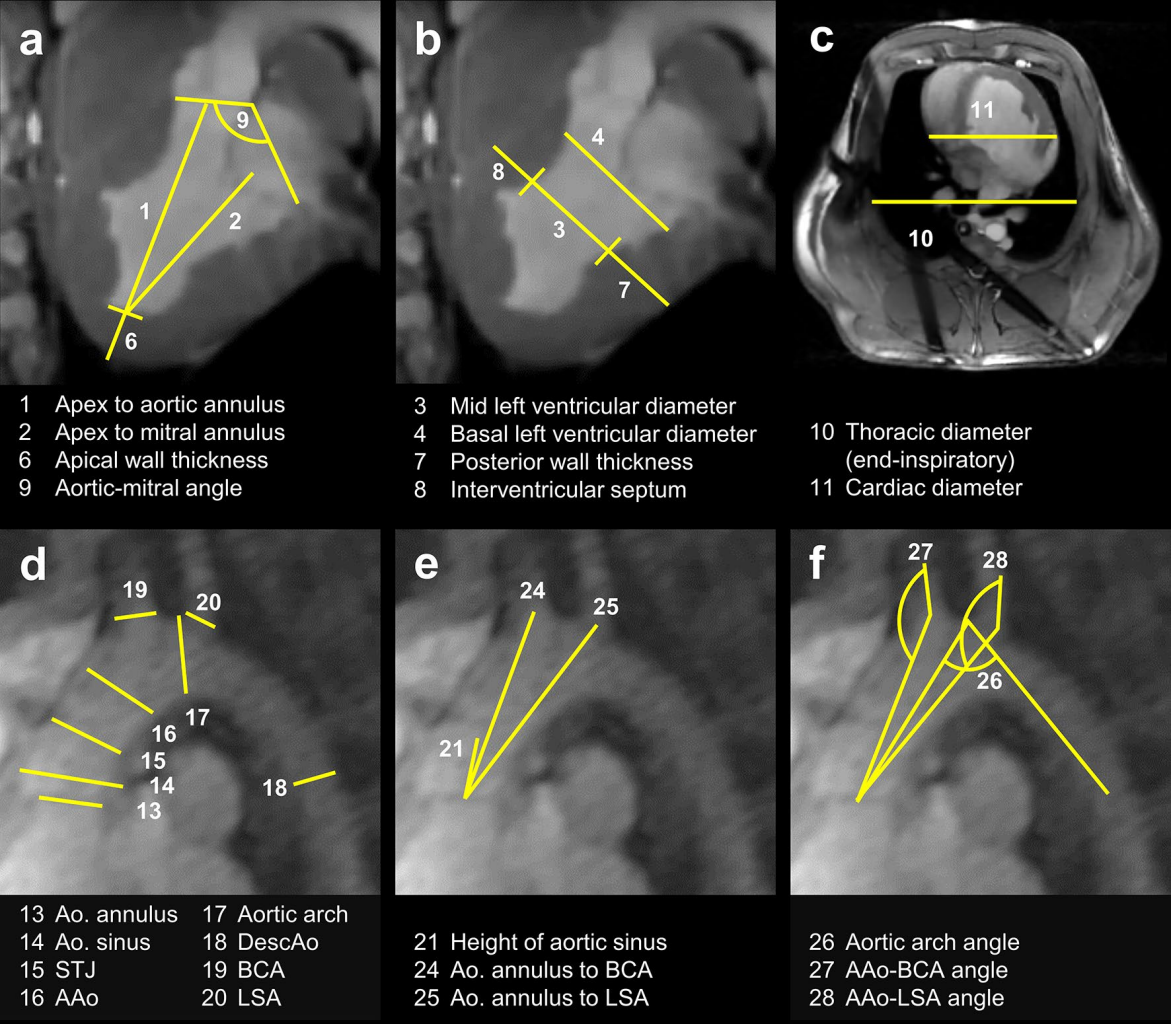
Porcine cardioaortic dimensions obtained from MRI. AAo, ascending aorta; Ao, aortic; BCA, brachiocephalic artery; DescAo, descending aorta; LSA, left subclavian artery; LV, left ventricle; STJ, sinotubular junction.

The web-based image analysis tool NORA (Medical Imaging Platform Project, University Medical Center Freiburg, Germany) was used to assess the cardioaortic geometry in pigs. The following distances (d/s, end-diastolic/systolic) were determined (Fig. 1a‒c): (1_d_) Apex to AV, (2_d_) apex to mitral valve (MV), (3_d/s_) mid and (4_d/s_) basal LV diameter, (6) apical and (7_d/s_) posterior wall thickness (PWT), (8_d/s_) interventricular septal thickness (IVST), (9_d_) aortic-mitral angle, (10) transverse end-inspiratory thoracic and (11) cardiac diameter. (5) Left ventricular sphericity was calculated as the ratio of LV length and diameter, defined as the distance from (2_d_) apex to MV and the (3_d_) end-diastolic mid-LV diameter. (12) Cardiothoracic ratio (CTR) was calculated as the ratio of the (11) cardiac and (10) thoracic diameter.

Moreover, the following aortic parameters were obtained (Fig. 1d‒f): Diameters of the (13) aortic annulus, (14) aortic sinus, (15) STJ, (16) tubular AAo, (17) aortic arch, (18) descending aorta (DescAo) at the height of the pulmonary bifurcation, proximal (19) BCA and (20) left subclavian artery (LSA). We also assessed the distances between the aortic annulus and (21) STJ, (22) left (LCA), and (23) right coronary artery (RCA), as well as (24) BCA and (25) LSA. To evaluate the endovascular access for coronary interventions and TAVI, we assessed the (26) angle of the aortic arch, measured between its highest point and the mid-AAo/DescAo lumen at the height of the pulmonary artery, as well as the angles between the aortic arch and the (27) BCA and (28) LSA. Moreover, the LV ejection fraction (LVEF) and LV volumes were determined.

### Statistical analysis

Metric variables are reported as means with standard deviation (SD). End-diastolic and end-systolic dimensions were compared by mean difference (MD). Differences between cardioaortic dimensions and male and female pigs were tested using a two-sided Student’s t-test. Pearson’s correlation coefficient was calculated to assess the correlation between cardioaortic dimensions and BW. A significance level of 5% and a confidential interval of 95% were applied. All calculations were performed with *R* (R Development Core Team, Vienna, Austria).

## Results

### Cardiac dimensions

**Figure 2:**
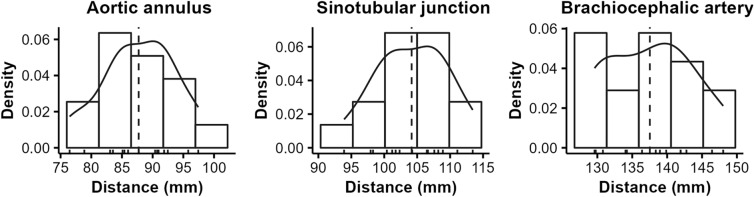
Longitudinal cardioaortic dimensions measured from the left ventricular apex. The distances between the apex and aortic annulus, sinotubular junction, and brachiocephalic artery differ between the animals by about 2 cm.

The porcine cardiothoracic dimensions derived from cMRI are summarized in Table 1. The distances from the LV apex to the AV and MV were 87.7 ± 5.9 and 81.1 ± 5.4 mm, respectively. The mid LV diameter was smaller than the basal one and decreased significantly from end-diastolic (50.5 ± 4.4 mm) to end-systolic (40.0 ± 4.2 mm) (Fig. 2a). The LV sphericity was 1.6 ± 0.1. The apical wall thickness varied substantially between the animals (range 3.3‒7.7 mm). The PWT increased from end-diastolic to end-systolic from 5.1 ± 0.8 to 7.7 ± 1.7 mm and the IVST from 5.8 ± 0.8 to 9.1 ± 1.7 mm. The aortic-mitral angle was 134.5 ± 8.9°, and the thoracic and cardiac diameters were 173.7 ± 11.7 and 98.1 ± 8.1 mm, respectively.

**Table 1:** Cardiac dimensions.

Cardiac dimensions	Mean ± SD
Lengths (mm)	
*1*_*d*_ Apex‒Aortic annulus	87.7 ± 5.9
*2*_*d*_ Apex‒Mitral annulus	81.1 ± 5.4
Diameters (mm)	
*3*_*d*_ LVEDD mid	50.5 ± 4.4
*3*_*s*_ LVESD mid	40.0 ± 4.2
*4*_*d*_ LVEDD basal	52.7 ± 3.4
*4*_*s*_ LVESD basal	40.2 ± 4.7
*5* LV sphericity	1.6 ± 0.1
Wall thickness (mm)	
*6* Apical wall	5.0 ± 1.0
*7*_*d*_ PWT ED	5.1 ± 0.8
*7*_*s*_ PWT ES	7.7 ± 1.7
*8*_*d*_ IVST ED	5.8 ± 0.8
*8*_*s*_ IVST ES	9.1 ± 1.7
Other	
*9* Aortic-mitral angle (°)	134.5 ± 8.9
*10* Thoracic diameter (mm)	173.7 ± 11.7
*11* Heart diameter (mm)	98.1 ± 8.1
*12* Cardiothoracic ratio	0.6 ± 0.1

ED: end-diastolic, ES: end-systolic, IVST: interventricular septal thickness, LV: left ventricle, LVEDD: left ventricular end-diastolic diameter, LVESD: left ventricular end-systolic diameter, PWT: posterior wall thickness, SD: standard deviation.

### Aortic dimensions

The dimensions of the thoracic aorta are summarized in Table 2. The diameters of aortic annulus, aortic sinus, and STJ were 19.3 ± 1.8, 26.2 ± 2.3, and 20.7 ± 2.3 mm, respectively. The diameter of the tubular AAo was 21.3 ± 2.3 mm. The aortic diameter decreased towards the DescAo. The two supra-aortic branches, BCA and LSA, revealed proximal diameters of 10.9 ± 1.3 and 8.8 ± 1.0 mm, respectively. The height of the aortic sinus was 16.5 ± 1.9 mm, and the ostium of the LCA was significantly closer to the aortic annulus than the RCA one (8.3 ± 1.3 *vs* 14.0 ± 1.2 mm, *p* < 0.05). Since BCA and LSA arise next to each other, the mean distances from the AV to the BCA and LSA differed by a few millimeters (49.8 ± 3.2 *vs* 58.4 ± 3.4 mm). The angle of the aortic arch was 71.9 ± 2.9°, and the AAo-BCA and AAo-LSA angles were 149.4 ± 6.6° and 144.7 ± 3.9°, respectively.

**Table 2:** Aortic dimensions.

Aortic dimensions	Mean ± SD
Diameters (mm)	
*13* Aortic annulus	19.3 ± 1.8
*14* Aortic sinus	26.2 ± 2.3
*15* Sinotubular junction	20.7 ± 2.3
*16* Ascending aorta	21.3 ± 2.3
*17* Aortic arch	22.6 ± 2.5
*18* Descending aorta	16.2 ± 1.4
*19* Brachiocephalic artery	10.9 ± 1.3
*20* Left subclavian artery	8.8 ± 1.0
Heights (mm)	
*21* Aortic sinus	16.5 ± 1.9
*22* LCA ostium	8.3 ± 1.3
*23* RCA ostium	14.0 ± 1.2
Lengths (mm)	
*24* Ao. annulus‒BCA	49.8 ± 3.2
*25* Ao. annulus‒LSA	58.4 ± 3.4
Angulation (°)	
*26* Aortic arch	71.9 ± 2.9
*27* Brachiocephalic artery	149.4 ± 6.6
*28* Left subclavian artery	144.7 ± 3.9

BCA: brachiocephalic artery, LCA: left coronary artery, LSA: left subclavian artery, RCA: right coronary artery, SD: standard deviation.

### Longitudinal dimensions

We determined the longitudinal dimensions between the LV apex and the (1) aortic annulus, STJ (1 + 21), and BCA (1 + 24). Figure 2 shows the distribution of all three lengths. As mentioned above, the distance from the LV apex to the aortic annulus was 87.7 ± 5.9 mm and ranged from 76.5 to 97.4 mm. The distances from apex to STJ and BCA were 104.2 ± 5.4 and 137.5 ± 6.1 mm. There was no relevant correlation between different longitudinal dimensions within the reported BW class.

### Left ventricular function

The LV function was characterized by an LVEF of 51.2 ± 9.8% as well as an LVEDV and LVESV of 84.7 ± 16.5 and 42.2 ± 13.2 mL (*MD* = 42.5 mL, stroke volume), respectively. The change in LV volume corresponded to a decrease of the basal and mid LV diameters by 12.6 and 10.5 mm, respectively.

### Comparison between male and female pigs

Comparing male and female pigs (weight 64.7 ± 6.2 *vs* 57.5 ± 6.2 kg, *MD* = 7.2 kg, *p* = 0.043), there were no significant differences in terms of cardioaortic dimensions, LVEF or stroke volume. However, male pigs revealed a smaller end-diastolic LV volume than females (75.6 ± 14.6 *vs* 92.6 ± 14.3 mL, *MD* = 17.1 mL, *p* = 0.041).

### Correlation between body weight and cardiac dimensions

**Figure 3:**
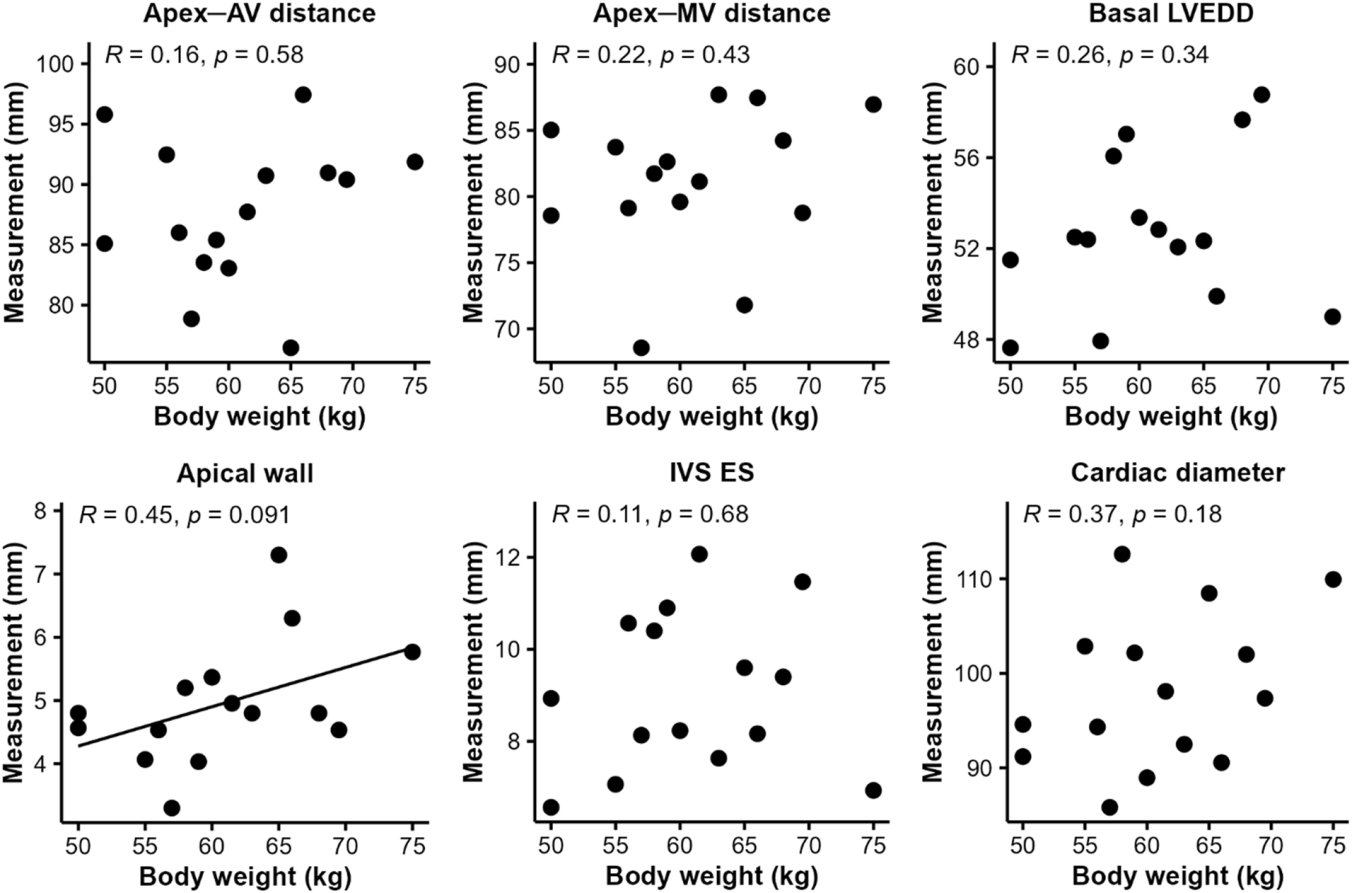
Correlation between body weight and cardiac dimensions in pigs. Apical wall thickness correlated moderately with body weight (*R* = 0.45, *p* = 0.091). All other parameters revealed a weak correlation (*R* < 0.40). AV, aortic valve; ES, end-systolic; IVS, interventricular septum; LVEDD, left ventricular end-diastolic diameter; MV, mitral valve.

A moderate but insignificant correlation was found between BW and apical wall thickness (*R* = 0.45, *p* = 0.091). All other cardiac parameters revealed no relevant correlation (Fig. 3).

### Correlation between body weight and aortic dimensions

**Figure 4:**
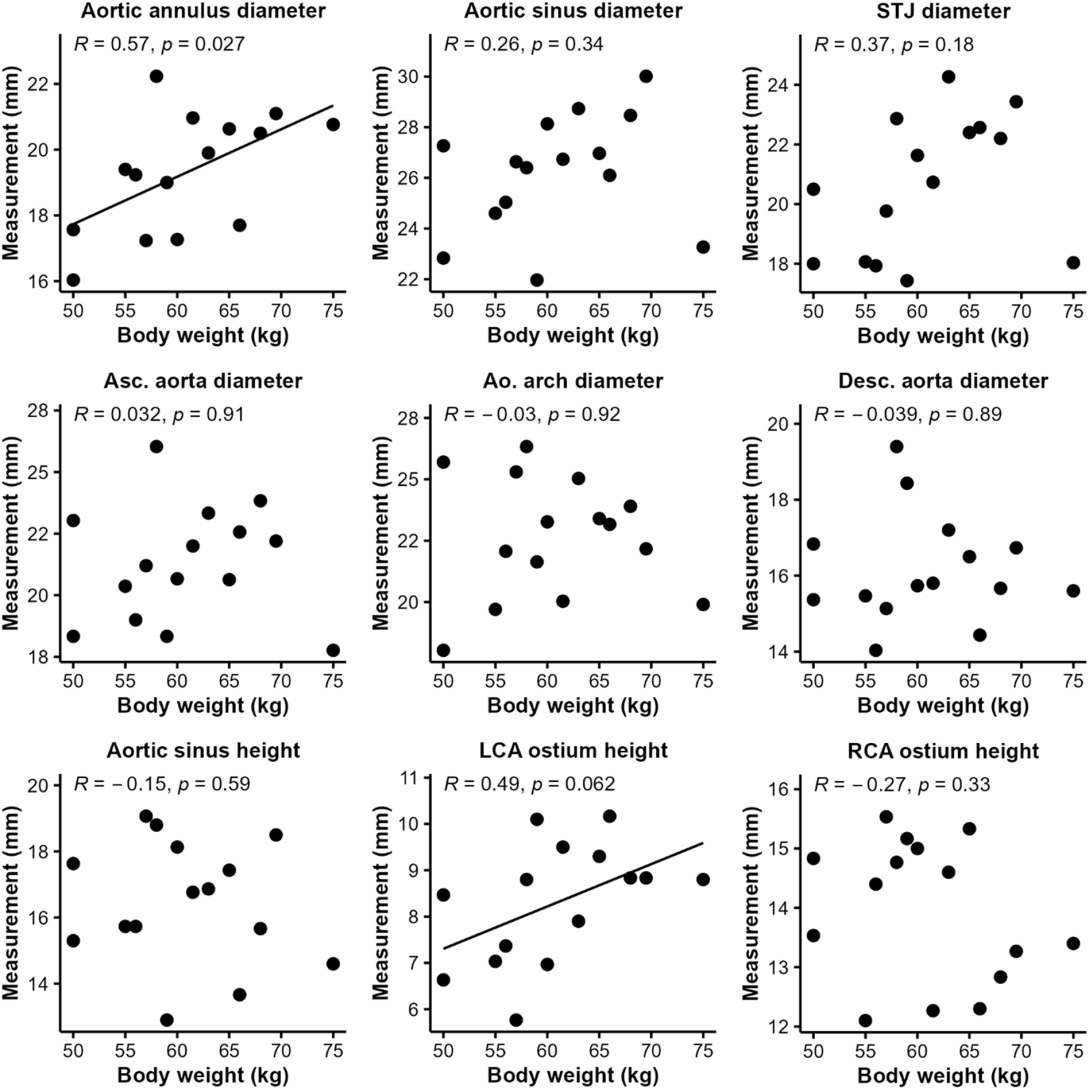
Correlation between body weight and aortic dimensions in pigs. Aortic annulus diameter and left coronary artery ostium height correlated moderately with body weight (*R* = 0.60 and 0.63). All other variables revealed only a weak correlation (*R* < 0.40). LSA, left subclavian artery; RCA, right coronary artery; STJ, sinotubular junction.

A moderate correlation was found between BW and the diameter of the aortic annulus (*R* = 0.57, *p* = 0.027) and BW and LCA ostium height (*R* = 0.49, *p* = 0.062). All other parameters revealed no relevant correlation with respect to BW (Fig. 4).

## Discussion

Pigs are commonly used in cardiovascular research and for medical device testing. Despite remarkable similarities between pigs and humans in cardiovascular anatomy and physiology, there are significant differences between both species^4,7,8^. Comprehensive knowledge of these differences and the cardioaortic dimensions is essential for planning and refining animal studies. Thus, such data allows optimal animal and device selection, thereby increasing animal safety during testing, reducing sample size and costs^4^.

Overall, we found only a few studies investigating the cardioaortic dimensions of domestic pigs. Paslawska et al*.* reported routine echocardiographic measurements in Polish Landrace pigs (*N* = 132, weight 20‒160 kg). A subgroup of animals (*N* = 25) with nearly the same weight range (50‒80 kg) revealed similar end-diastolic but smaller end-systolic LV diameters and a greater increase in IVST and PWT during systole^17^. Since the pigs in our study underwent temporary coronary balloon occlusion just before the cMRI, there might have been a persisting impairment of LV function leading to slight differences. Wang et al*.* determined the dimensions of the aortic root and thoracic aorta from explanted pig hearts (*N* = 8, mean weight 89 kg). The aortic sinus and aortic arch diameters reported by them were smaller than in our cohort (5.7 and 3.6 mm less), whereas all other parameters were comparable^18^. Data obtained from CTA on a series of Swiss Large White pigs (*N* = 24, overall mean weight 79 kg) were published by Lipiski et al. Pigs from the lowest of three BW classes (*N* = 8, weight 53 kg) revealed aortic dimensions comparable to our results. The authors also assessed the correlation between BW (weight range 50‒110 kg) and various dimensions to predict the dimensions of the AV for TAVI procedures. With increasing BW, Swiss Large White pigs revealed an increased LV outflow tract and AV diameter, as well as a longer AAo and higher RCA ostium. However, there was no correlation between BW and the aortomitral angle or the height of the LCA^4^. Although BW is an important predictor for various dimensions, there are many more determinants, including sex, body height, and age^19,20^.

The LV dimensions among pigs and humans differ in various ways. Despite similar LV end-diastolic diameters, pigs appear to have smaller LV volumes^21^, which might be explained by coarser LV trabeculations and the Valentine heart shape^7^. Moreover, the porcine LCA ostium arises closer to the aortic annulus than in humans^22^. Therefore, referring to TAVI procedures in pigs, lower and self-expandable valves should be preferred to prevent coronary occlusion during TAVI procedures^23^. For cardiac catheterization, the smaller angle between the porcine LCA and RCA (90°) and the slightly different direction of the left anterior descending coronary should be considered^8^.

In general, pigs appear to have smaller AV and aortic diameters than humans. According to cMRI studies, men and women reveal a mean systolic AV diameter of 36 and 34 mm, respectively^24^. The smaller porcine aortic diameter and curvature impact the design and choice of interventional instruments for animal studies. Considering the relatively small aortic arch angle, catheters and guidewires must withstand higher bending forces when accessing the coronary arteries or the LV in pigs. According to Manole et al*.*, humans also reveal greater BCA and LSA diameters as supra-aortic branches^25^, which are relevant for subclavian and carotid vascular access.

In our study, cMRI volumetry revealed an LVEF of about 50% and an LVEDV and LVESV of about 85 and 45 mL, respectively. These findings are consistent with data from Van Essen et al*.* for female Yorkshire Landrace pigs (*N* = 10, weight < 75 kg), who reported a cardiac output of 4.3 L/min, an LVEF of 61% (3D epicardial echocardiography) and an LVEDV of 81 mL (conductance catheter). Analyzing higher BW classes (total *N* = 31), the same study showed that the porcine cardiac output increases logarithmically with higher BW. A larger and more muscular LV (increased LV weight) produces a larger stroke volume while heart rate and LVEF remain stable^26^. Nevertheless, the pigs from our study revealed smaller LV volumes^27^ and a smaller LVEF^21^ than humans.

Depending on the application, different cardioaortic dimensions may be relevant. The differences in cardiac size between pigs and humans determine practical details of cardiovascular procedures, such as the preferred vascular access. Smaller vascular diameters impair the endovascular delivery of catheters and medical devices. Reduced aortic root and the AAo diameters should be considered for TAVI procedures and aortic stent graft implantation. Oversized stent grafts might favor the formation of aortic aneurysms and, eventually, aortic rupture. The diameters of the great vessels are critical for porcine-to-human xenotransplantations to avoid size mismatches between porcine donor and human recipient^8^. Referring to the pioneering study from Griffith et al*.*^28^, the dimensions of the genetically modified porcine heart, particularly the diameters of the AAo and pulmonary artery, were significantly smaller than the ones from the human recipient. Therefore, the anastomoses had to be sutured asymmetrically^8^.

For transapical LVAD implantation in pigs, it is also important to consider the apical wall thickness, which may vary substantially from pig to pig. We assume this is due to the densely trabeculated inner LV surface, which may also compromise the accuracy and reproducibility of MRI-derived measurements. In LVAD systems with transventricular outflow graft across the AV, the distances from the LV apex to the STJ and BCA define the outflow length. In case of a length mismatch, especially in pigs with short AAo, retrograde graft dislocation through the AV or brachiocephalic malperfusion may occur. For optimal device attachment to the LV apex, a customized sewing ring with improved fitting to the more conical porcine apex should be considered. During LVAD support and mechanical LV unloading, the smaller porcine LV chamber might predispose suction events. Although most in vivo studies on mechanical circulatory support devices are performed in young, healthy animals^1^, such tests should ideally be performed in a heart failure model (e.g., rapid pacing, coronary embolization, coronary ligation^29^), favoring additional dimensional changes.

Due to the small sample size, this study has an explorative character. The BW distribution and the number of animals for different weights are relatively small. Therefore, a statistically significant conclusion regarding the correlation to BW cannot be drawn from the presented data. In future studies, cMRI-derived measurements should be performed on more animals, potentially of different breeds and BWs, to assess the correlation between BW and different cardioaortic dimensions appropriately. Moreover, all animals underwent temporary coronary occlusion, which is likely to impair LV geometry and function. Although the systolic function remained normal in our study, and there was no evidence of myocardial edema, wall thickening, or segmental wall motion abnormalities, other secondary alterations cannot be excluded. We assume that the temporary coronary occlusion either did not cause any myocardial damage or that the damage would have become apparent later on. Last, the imaging quality might be improved by optimizing cMRI acquisition parameters to precisely assess the complex cardiac geometry.

## Conclusions

Cardiac MRI in pigs allows for obtaining specific cardioaortic dimensions required for developing and pre-clinical testing of medical devices. German Landrace pigs reveal relatively small LV chambers, which may affect the testing of LVAD systems developed for patients with (mainly) dilated cardiomyopathy and enlarged cardiac dimensions. Furthermore, the porcine aortic root and thoracic aorta reveal smaller diameters, and the AAo is much shorter than in humans. Our data indicate no significant differences between male and female pigs with respect to their cardioaortic dimensions. Our results help researchers and engineers optimize medical devices for pre-clinical testing in pigs and support animal and device selection.

## Data Availability

The datasets used and analyzed during the current study are available from the corresponding author upon reasonable request.
